# Effect of erythrophagocytosis-induced ferroptosis during angiogenesis in atherosclerotic plaques

**DOI:** 10.1007/s10456-023-09877-6

**Published:** 2023-04-29

**Authors:** Pauline Puylaert, Lynn Roth, Melissa Van Praet, Isabel Pintelon, Catalina Dumitrascu, Alexander van Nuijs, Greta Klejborowska, Pieter-Jan Guns, Tom Vanden Berghe, Koen Augustyns, Guido R. Y. De Meyer, Wim Martinet

**Affiliations:** 1 Laboratory of Physiopharmacology, University of Antwerp, Universiteitsplein 1, B-2610 Antwerp, Belgium; 2 Infla-Med Centre of Excellence, University of Antwerp, Antwerp, Belgium; 3 Laboratory of Cell Biology and Histology, University of Antwerp, Antwerp, Belgium; 4 Toxicological Centre, University of Antwerp, Antwerp, Belgium; 5 Laboratory of Medicinal Chemistry, University of Antwerp, Antwerp, Belgium; 6 VIB-UGent Center for Inflammation Research, Ghent, Belgium; 7 Department of Biomedical Molecular Biology, Ghent University, Ghent, Belgium; 8 Department of Biomedical Sciences, University of Antwerp, Antwerp, Belgium

**Keywords:** Atherosclerosis, Intraplaque angiogenesis, Hemorrhage, Erythrophagocytosis, Ferroptosis

## Abstract

Intraplaque (IP) angiogenesis is a key feature of advanced atherosclerotic plaques. Because IP vessels are fragile and leaky, erythrocytes are released and phagocytosed by macrophages (erythrophagocytosis), which leads to high intracellular iron content, lipid peroxidation and cell death. In vitro experiments showed that erythrophagocytosis by macrophages induced non-canonical ferroptosis, an emerging type of regulated necrosis that may contribute to plaque destabilization. Erythrophagocytosis-induced ferroptosis was accompanied by increased expression of heme-oxygenase 1 and ferritin, and could be blocked by co-treatment with third generation ferroptosis inhibitor UAMC-3203. Both heme-oxygenase 1 and ferritin were also expressed in erythrocyte-rich regions of carotid plaques from *ApoE*^*−/−*^ *Fbn1*^*C1039G+/−*^ mice, a model of advanced atherosclerosis with IP angiogenesis. The effect of UAMC-3203 (12.35 mg/kg/day) on atherosclerosis was evaluated in *ApoE*^*−/−*^ *Fbn1*^*C1039G+/−*^ mice fed a western-type diet (WD) for 12 weeks (n = 13 mice/group) or 20 weeks (n = 16–21 mice/group) to distinguish between plaques without and with established IP angiogenesis, respectively. A significant decrease in carotid plaque thickness was observed after 20 weeks WD (87 ± 19 μm vs. 166 ± 20 μm, *p* = 0.006), particularly in plaques with confirmed IP angiogenesis or hemorrhage (108 ± 35 μm vs. 322 ± 40 μm, *p* = 0.004). This effect was accompanied by decreased IP heme-oxygenase 1 and ferritin expression. UAMC-3203 did not affect carotid plaques after 12 weeks WD or plaques in the aorta, which typically do not develop IP angiogenesis. Altogether, erythrophagocytosis-induced ferroptosis during IP angiogenesis leads to larger atherosclerotic plaques, an effect that can be prevented by ferroptosis inhibitor UAMC-3203.

## Introduction

Advanced human atherosclerosis is characterized by unstable plaques occupying large- and medium-sized arteries. The stability of the plaque determines its fate, either toward a low-risk lesion defined by a thick fibrous cap and a relatively small necrotic core, or toward a high-risk lesion with a highly inflammatory necrotic core and a thin fibrous cap prone to rupture. Intraplaque (IP) angiogenesis is an important feature of advanced human plaques. Because IP neovessels are often leaky, they facilitate IP hemorrhages and contribute significantly to plaque instability by promoting the entry of pro-inflammatory cells, erythrocytes and low-density lipoproteins in the plaque microenvironment [1, 2]. Interestingly, macrophages surrounding IP neovessels are activated and show signs of platelet- and erythrophagocytosis [1, 3, 4]. The accumulation of intracellular hemoglobin and iron after erythrophagocytosis may act as a catalyst in the formation of free radicals, contributing to lipid peroxidation, modifications of low-density lipoproteins (LDL) and cell death [1, 4].

Ferroptosis is a recently identified form of necrotic cell death that is dependent on intracellular iron and characterized by lipid peroxidation [5]. Canonical induction of ferroptosis involves glutathione peroxidase 4 (GPX4), either by direct inhibition of GPX4 or through depletion of its substrate glutathione [5, 6]. Intracellular iron levels are tightly regulated by a fine interplay of several iron-binding proteins. Free Fe(II) resides in a labile iron pool (LIP), which is tightly controlled by ferritin that oxidizes Fe(II) and stores it as Fe(III). Iron export to and transport in the circulation are regulated by ferroportin and transferrin, respectively. Heme-oxygenase 1 (HMOX1) is responsible for the catabolism of heme into biliverdin, carbon monoxide and Fe(II), and is highly relevant in the context of erythrophagocytosis. Non-canonical induction of ferroptosis occurs when the finely regulated iron balances are disrupted, for example by overloading cells with iron (e.g., derived from hemoglobin), resulting in growth of the LIP beyond the buffering capacities of ferritin [7]. Possible mechanisms include excessive activation of HMOX1, decreased ferroportin expression and increased transferrin expression [7, 8]. Interestingly, ferroptosis can be pharmacologically blocked, e.g. by Ferrostatin-1 (Fer-1). The anti-ferroptosis activity of Fer-1 and analogues can be explained by the fact that they act as potent radical trapping agents, especially in lipid bilayers [9, 10].

IP angiogenesis and hemorrhages in advanced human plaques contribute to plaque instability and necrotic core formation, but the exact mechanisms are not fully understood [1]. Macrophages surrounding IP hemorrhage phagocytose erythrocytes, which in turn leads to HMOX1 activation and high intracellular levels of biliverdin and iron [3, 4, 11]. Excessive HMOX1 activation protects against heme-induced oxidative damage [12], but also serves as a trigger for non-canonical ferroptosis induction [7]. Therefore, we hypothesize that IP hemorrhages trigger ferroptosis in macrophages after erythrophagocytosis. Macrophages undergoing ferroptotic cell death lose membrane integrity and leak a myriad of molecules, many of which fall into the category of damage-associated molecular patterns (DAMPs). Some well-established DAMPs include mitochondria-derived N-formylated peptides, DNA and RNA, the nuclear protein HMGB1, histones, actin, calcium-binding S100 proteins, heat-shock proteins, ATP and uric acid [13]. DAMPs are potent inducers of inflammation through activation of pattern-recognition receptors such as toll-like receptors and NOD-like receptors. Necrotic cells may also release pre-stored pro-inflammatory cytokines and chemokines, which may directly or indirectly recruit phagocytes to the vicinity [13]. The deposition of necrotic debris may subsequently contribute to the growth of the necrotic core and destabilization of the plaque. To test this hypothesis, erythrophagocytosis-induced ferroptosis was characterized both in vitro and in vivo in *ApoE*^*−/−*^ *Fbn1*^*C1039G+/−*^ mice, a model of advanced atherosclerosis showing IP angiogenesis and hemorrhage [14, 15]. Furthermore, the effect on atherogenesis of pharmacological inhibition of ferroptosis with UAMC-3203, a novel Ferrostatin-1 (Fer-1) analogue with increased stability and potency, was evaluated [10, 16].

## Materials and methods

### Compounds

1 S,3R-RSL3 (Tocris: 6118), ferric ammonium citrate (Sigma Aldrich), UAMC-3203 and UAMC-3206 (provided by the Laboratory of Medicinal Chemistry, University of Antwerp, Belgium) [10], zVAD-fmk (Enzo Life Sciences: ALX-260-020-M001), Necrostatin-1s (Nec-1s, Abcam: ab221984), ferrostatin-1 (Fer-1, Sigma Aldrich: SML0583), Deferoxamine (DFO, Sigma Aldrich: D9533), vitamin E (*α*-tocopherol; Sigma Aldrich: T3251-5G).

### Mouse strains

**Fig. 1:**
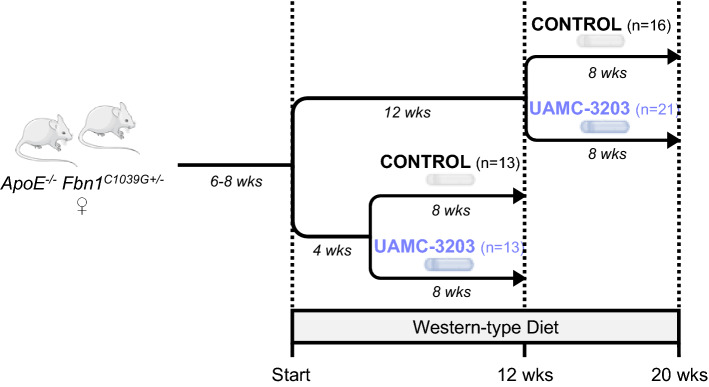
Atherosclerosis study with UAMC-3203. Treatment regimen of *ApoE*^*−/−*^ *Fbn1*^*C1039G+/−*^ mice with western-type diet (WD) and osmotic minipumps containing saline solution or UAMC-3203

Wild-type (WT) mice (C57BL/6J, Jackson Laboratories) and *GPX4*^*Tg/+*^ mice [17] overexpressing GPX4 were used for isolation of primary bone marrow-derived macrophages (BMDMs). To study the in vivo *stability of UAMC-3203*, female WT mice (3 months of age, C57BL/6J, Jackson Laboratories) were treated with 12.35 mg/kg/day UAMC-3203 or saline solution via subcutaneously implanted osmotic minipumps (Alzet pump type 1004, Charles River) for 2 weeks. For atherosclerosis studies, female *ApoE*^*−/−*^ *Fbn1*^*C1039G+/−*^ mice (aged 6–8 weeks) were fed a Western-type diet (WD; TD.88137 supplemented with 21% fat and 0.2% cholesterol, Envigo) for 12 or 20 weeks. Mice were divided over 2 treatment regimens with their respective controls (Fig. 1). After 4 or 12 weeks WD, mice were treated with 12.35 mg/kg/day UAMC-3203 or saline solution via subcutaneous osmotic minipumps (Alzet pump type 1004, 4 weeks, Charles River) for 8 weeks in total. The first osmotic minipump, containing saline solution or UAMC-3203, was replaced after 4 weeks by a second minipump to reach a total of 8 weeks of treatment in all groups. Animals were housed in a temperature-controlled room with a 12 h light/dark cycle and had free access to water and food. Cases of sudden death and head tilt were documented. The subcutaneous implantation of osmotic minipumps was performed under anesthesia (isoflurane 3–4% and O_2_ in a ventilated gas mixture) and on a heating element to prevent hypothermia. After shaving and disinfecting the site of implantation, an incision was made on the upper dorsal side. A subcutaneous pocket was made using a hemostat. Subsequently, the filled pump was inserted in the pocket (on opposite sides for pump 1 and pump 2) and the wound was closed with interrupted sutures (size 5 − 0 Ethilon™ II suture on a 19 mm 3/8ths of a circle cutting needle). At the end of the study, an overdose of sodium pentobarbital (250 mg/kg, *i.p.*) was administered and tissues were collected for histological analysis. Further, blood samples were collected via the retro-orbital plexus. Plasma levels of total cholesterol were measured using a commercially available kit (Randox laboratories).

### Histological analyses

The thoracic and abdominal aorta were stained *en face* with Oil Red O to determine lipid burden. The proximal ascending aorta and right common carotid artery (RCCA) were fixed in 4% formaldehyde (pH 7.4) for 24 h, dehydrated overnight in 60% isopropanol and subsequently embedded in paraffin. Serial cross-sections (5 μm) of the proximal ascending aorta and serial longitudinal sections (5 μm) of the RCCA were prepared at random for histological analyses. Atherosclerotic plaque thickness and necrotic core area (defined as acellular areas with a threshold of 3000 µm^2^) were analyzed on hematoxylin-eosin (H&E) stained sections. Plaque thickness was measured on 10 random locations over the full length of a longitudinal section of the RCCA and mean plaque thickness was calculated. Total collagen was measured on Sirius red stained sections. For immunohistochemistry, the following antibodies were used: anti-Mac3 (BD Pharmingen, 550292); anti-α-smooth muscle actin (α-SMA; Sigma, 12547), anti-heme-oxygenase 1 (HMOX1; Enzo Life Sciences, ADI-SPA-896), anti-Ferritin heavy chain (FTH; Abcam, ab185781) and anti-TER119 (BD Pharmingen, 550565). Immunohistochemical staining for HMOX1, FTH and TER119 were always performed on serial sections. Images were acquired with an Olympus BX43 microscope. Plaques were manually delineated in Image J software (National Institutes of Health) to establish the region of interest (ROI). Further analyses within the ROIs were performed using color thresholding or manual counting (apoptotic cells). For all histological analyses, the observer was blinded to the received treatment (UAMC-3203 or saline).

### Cell culture

Bone marrow-derived macrophages (BMDMs) were isolated by flushing femurs of C57BL/6J mice with RPMI medium containing L-glutamine and HEPES (Gibco Life Technologies) and supplemented with 10 U/mL heparin (Sigma). The cell suspension was pipetted through a 40 μm cell strainer (Greiner Bio-One: Easystrainer™ 542,040), washed twice and resuspended in RPMI medium with Glutamax™. The RPMI medium was supplemented with 10% FBS, 1% penicillin-streptomycin (10.000 U/mL, Gibco Life Technologies) and 0.2% polymyxin B (10.000 U/mL, Fagron) and 15% L-cell conditioned medium (LCCM) containing monocyte colony stimulating factor (M-CSF).

Recently expired (up to 5–7 days) concentrates of human erythrocytes or blood platelets were obtained from the Belgian Red Cross. Erythrocyte concentrates were stored at 4 °C for maximum 2 months. Before use, erythrocyte concentrates were freshly diluted 1:1 with PBS, centrifuged for 5 min at 201×*g* and washed with PBS. The number of intact erythrocytes was counted to ensure that macrophages were treated with the same number of erythrocytes in each experiment. Platelet concentrates were kept shaken at room temperature for maximum 2 months and were centrifuged for 10 min at 1258×*g* and washed twice with PBS. Erythrocyte concentrates from different donors and pooled platelet concentrates were used between experiments to account for the natural variation that exists between humans.

At day 7, 0.5 × 10^6^ BMDMs were incubated with 4 × 10^7^ erythrocytes (BMDM:erythrocyte ratio = 1:80) for 0.5, 1, 2 and/or 4 h, depending on the experimental set-up. BMDMs treated with vehicle (DMEM supplemented with 10% FBS, 1% penicillin-streptomycin and 0.2% polymyxin B) or with 10^8^ platelets (BMDM:platelet ratio = 1:200) served as controls. After phagocytosis of erythrocytes or platelets, BMDMs were washed twice with PBS to remove free erythrocytes or platelets. Remaining erythrocytes in the culture medium or attached to (though not internalized by) BMDMs were removed with Erythrocyte Lysis Buffer (Abcam, ab204733). Finally, BMDMs were detached with 0.25% trypsin-EDTA (Thermo Fischer Scientific, 25200072) and analyzed by flow cytometry using a BD Accuri C6 flow cytometer.

To evaluate cell viability, a neutral red assay was performed as previously described [18]. Briefly, cells were incubated with 0.1% neutral red solution at 37 °C in 95% air/5% CO_2_. Subsequently, cells were washed with PBS and neutral red was extracted by adding 0.05 M NaH_2_PO_4_ in 50% ethanol. After 3 min, optical density was read at 540 nm using a microplate reader.

### Flow cytometry

All sample analyses were performed on a BD accuri C6 flow cytometer. For each measurement, a gating strategy was applied based on fluorophore minus one (FMO) controls. Because both the treatment of BMDMs and the combination of fluorophores can result in background signals, there was always one FMO control per treatment per fluorophore included. Thus, for each treatment replicates were included to serve as FMO controls. FMO controls were treated in the same way as the samples and stained with all fluorophores except for the one they are controlling for. Debris was always gated out and at least 20,000 cells were measured.

#### Analysis of macrophage surface markers

Detached BMDMs were first treated with mouse anti-CD16/CD32 antibody solution (eBioscience, 14-0161-85; 1:50 dilution in FACS buffer [PBS with 0.1% bovine serum albumin and 0.05% sodium azide]) for 10 min at 4 °C to block nonspecific binding sites. After centrifugation (5 min, 201×*g*), BMDMs were resuspended in mouse anti-F4/80-FITC (Biolegend, 123,108; 1:50 dilution in FACS buffer) or mouse anti-F4/80-AF647 (Biolegend, 123,122; 1:500 dilution in FACS buffer) antibody solutions and incubated for 30 min at 4 °C.

#### Quantification of phagocytosis and cell death

Erythrocytes and platelets were stained with Cell Trace Far Red (CTFR; Thermo Fischer Scientific, C34564) according to the manufacturer’s protocol. Briefly, erythrocytes and platelets were counted and diluted in PBS. Subsequently, 1:1000 or 1:10,000 CTFR was added to the erythrocytes and platelets, respectively, and incubated for 20 min at 37 °C. After addition of an equal volume of DMEM, erythrocytes and platelets were centrifuged at 201×*g* and 1258×*g*, respectively, and resuspended in DMEM. Detached BMDMs were blocked and stained with F4/80-FITC macrophage surface marker as described above. Thereafter, BMDMs were washed once with FACS buffer. Propidium iodide (PI) was added to the samples just before measuring. The following gating strategy was used to identify macrophages with phagocytosed erythrocytes or platelets and dead/dying macrophages: F4/80-FITC positive in FL-1 channel for macrophages; F4/80-FITC positive in FL-1 channel and CTFR-positive in FL-4 channel for macrophages with phagocytosed CTFR-labelled erythrocytes or platelets; F4/80-FITC positive in FL-1 channel and PI-positive in FL-2 channel for dead/dying macrophages.

#### Quantification of lipid peroxidation

Detached BMDMs were incubated with 5 µM Bodipy C11 581/591 (Thermo Fischer Scientific, D3861) solution in FACS buffer for 30 min at 37 °C. Next, BMDMs were washed once with PBS and subsequently blocked for nonspecific binding and stained with F4/80-AF647 macrophage surface marker, as described above. After one additional washing step with FACS buffer, samples were immediately measured. The following gating strategy was used to characterize lipid peroxidation in macrophages: F4/80-AF647 positive in FL-4 channel for macrophages; F4/80-AF647 positive in FL-4 channel and Bodipy C11 positive in FL-1 channel for oxidized phospholipid (PL) fraction in macrophages; F4/80-AF647 positive in FL-4 channel and Bodipy C11 positive in FL-2 channel for reduced PL fraction in macrophages. The ratio of the mean fluorescence in FL-1 channel over the mean fluorescence in FL-2 channel was calculated to quantify lipid peroxidation. Positive controls consisted of BMDMs treated with 1 S,3R-RSL3.

#### Quantification of labile iron pool

Detached BMDMs were resuspended in 5 µM FeRhoNox-1 (Sigma-Aldrich, SCT030) and incubated for 1 h at 37 °C. After two washing steps with PBS, samples were immediately measured and labile iron was quantified in the FL-2 channel. Positive controls consisted of BMDMs treated with 1 S,3R-RSL3 and ferrous sulfate heptahydrate.

#### Quantification of apoptosis

Detached BMDMs were fixed with 4% paraformaldehyde for 1 h at room temperature. After washing with PBS, BMDMs were permeabilized with 0.1% Triton X-100 in 0.1% sodium citrate solution for 2 min on ice. BMDMs were washed again and incubated with TUNEL reaction mixture using an in situ cell death detection kit (Roche, fluorescein, 1168479591) for 1 h at 37 °C. The samples were washed twice with FACS buffer and measured in the FL-1 channel. Positive controls consisted of BMDMs treated with TNFα and cycloheximide.

### Confocal microscopy

Erythrocytes were stained with CTFR as described above. After phagocytosis, calcein green AM was added to the samples to stain BMDMs. Images were obtained using a microlens-enhanced dual spinning disk confocal microscope (UltraVIEW VoX; PerkinElmer) equipped with solid state 480 and 640 nm diode lasers for excitation of calcein green AM and CTFR, respectively. Images were processed using the reconstruction facilities of Volocity software (PerkinElmer).

### Western blotting

Cells were lysed with Laemmli sample buffer (Bio-rad, 1610737) containing β-mercaptoethanol (Sigma-Aldrich) and boiled for 5 min. To separate nuclear and cytosolic fractions, a nuclear extraction kit (Abcam, ab113474) was used following the manufacturer’s protocol. Samples were loaded on 4–12% Bis-Tris gels (Life Technologies, NW04125BOX) for electrophoresis and transferred to Immobilon-FL PVDF membranes (Millipore, IPFL00010) according to standard procedures. Subsequently, membranes were blocked for 1 h in Odyssey Li-COR blocking buffer. After blocking, membranes were probed overnight at 4 °C with primary antibodies diluted in Odyssey Li-COR blocking buffer followed by 1 h incubation with IRDye-labeled secondary antibodies at room temperature. Membranes were visualized with an Odyssey SA infrared imaging system (Li-COR Biosciences). The following primary antibodies were used: mouse anti-HMOX1 (Abcam, ab13248), rabbit anti-FTH1 (Abcam, ab183781), rabbit anti-GPX4 (Abcam, ab125066) and mouse anti-β-actin (Abcam, ab8226). IRDye® 800CW goat anti-rabbit (Li-Cor, Li 926-32211) or IRDye® 680RD goat anti-mouse (Li-Cor, LI 926-68070) were purchased from Li-COR Biosciences.

### Liquid chromatography-tandem mass spectrometry (LC-MS/MS)

A LC-MS/MS method was optimized and validated for the determination of UAMC-3203, as described previously [19]. UAMC-3203 detection in plasma, and tissue homogenates (lysed in PBS using a Precellys 24 Tissue Homogenizer (Bertin Instruments)) was performed on an Agilent 1200 series LC system connected to a 6410 triple quadrupole mass spectrometer from Agilent Technologies with electrospray ionization (ESI) interface operated in positive ionization mode. Chromatographic separation was carried out on a Kinetex Biphenyl column (100 × 2.1 mm, 2.6 μm; Phenomenex). The mobile phase consisted of (A) ultrapure water with 0.1% formic acid and (B) acetonitrile/ultrapure water (90/10) with 0.1% formic acid, in gradient at 0.3 mL/min. The ESI source parameters were gas temperature 350 °C, gas flow 10 L/min, nebulizer pressure 35 psi, and capillary voltage 4000 V. Data acquisition was done in multiple reaction monitoring mode (MRM). Confirmation of UAMC-3203 was done using three MRM transitions; the most abundant transition was used as a quantifier (Q) and the other two were used as a qualifier (q). Qualifier/quantifier ratios (q/Q) were calculated for each sample and had to be within ± 20% of the q/Q ratio observed in the calibrators. In addition, the retention time of the compound in samples could not deviate > 10% of the retention time observed in the calibrators.

UAMC-3203 and nordiazepam-D_5_ (Cerilliant Corporation) as internal standard (IS) were diluted in LC-MS grade methanol (Fisher Scientific). A volume of 100 µL sample was spiked with 20 µL IS (200 ng/mL), followed by the addition of 150 µL acetonitrile for plasma. For tissue homogenates, 500 µL acetonitrile with 0.1% formic acid was added. Subsequently, the mixture was vortexed (2 min) and centrifuged (10 min, 9168×*g* for plasma, 17,968×*g* for tissue). The supernatant of plasma was then transferred to a 2 mL tube with a 0.20 μm centrifugal filter (VWR). The supernatant of tissue was evaporated under a stream of nitrogen at 40 °C, reconstituted in 100 µL acetonitrile/ultrapure water (90/10) with 0.1% formic acid, and transferred to a 2 mL tube with a 0.20 μm centrifugal filter. All samples were then centrifuged (5 min 9168×*g* for plasma, 10 min 17,968×*g* for tissue), after which the final extract was transferred to an autosampler vial with a glass insert. Seven-level calibration curves were prepared in blank mouse plasma, covering a linear range from 10 to 700 ng/mL. Five-level calibration curves were prepared in blank homogenized mouse tissue matrix covering a linear range from 20 to 4000 ng/mL. The measured concentrations in ng/mL were further normalized using the weight of the tissue used for homogenization to obtain final concentrations of UAMC-3203 expressed in µg/g.

### Statistical analyses

Statistical analyses were performed using Graphpad Prism 9 and SPSS software (version 27, SPSS Inc.). All data are expressed as the mean ± SEM. Datapoints in the graphs represent n samples from independent experiments or individual mice (i.e. biological repeats). Statistical tests and number of replicates are specified in the text and figure legends. If a two-way ANOVA was used, a significant main effect was further analyzed by performing a simple main effect analysis, including Bonferroni correction for multiple comparisons. Differences were considered significant when p < 0.05.

## Results

### Erythrophagocytosis induces labile iron accumulation, lipid peroxidation and cell death in BMDMs

**Fig. 2:**
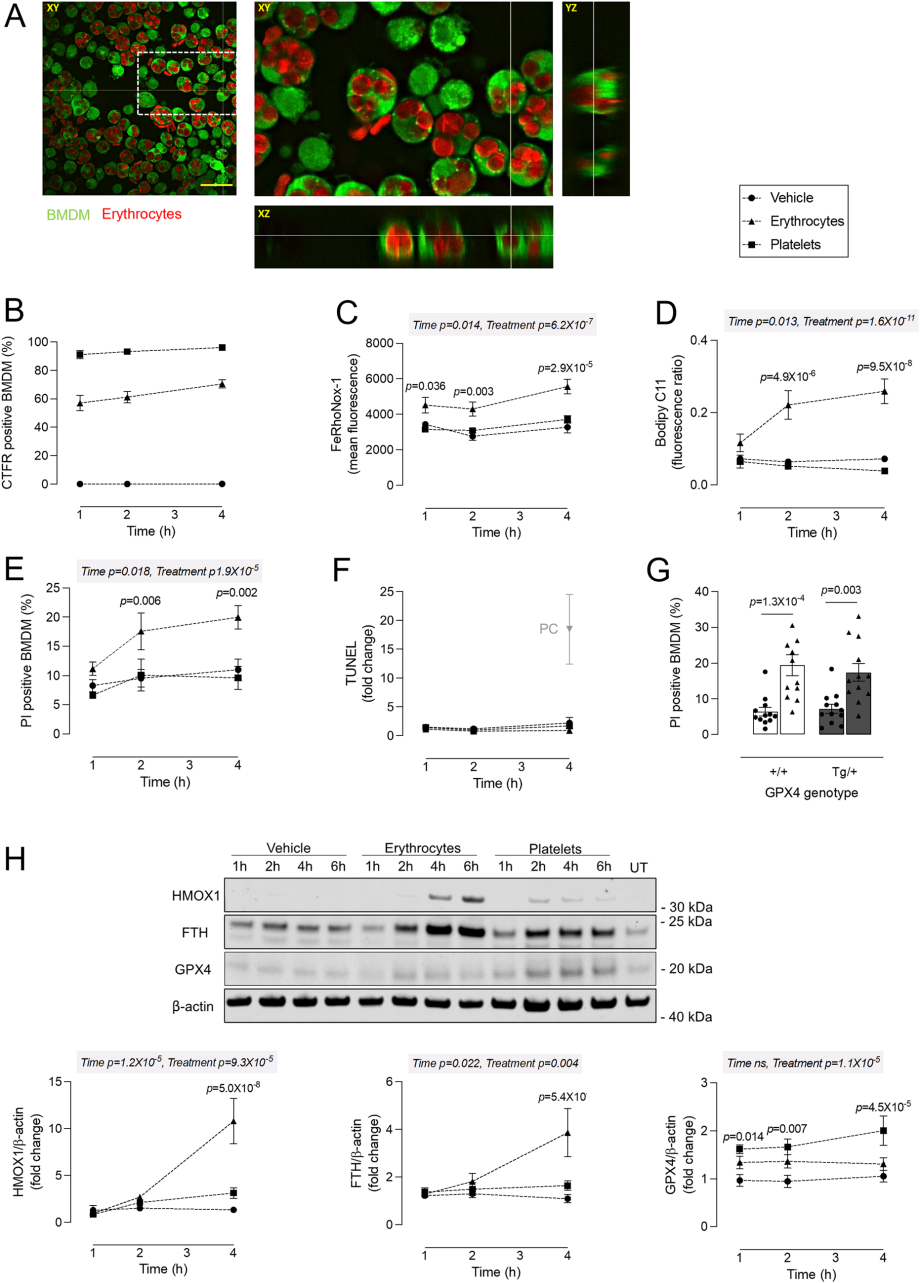
Erythrophagocytosis induces labile iron accumulation, lipid peroxidation and cell death in BMDMs. BMDMs were treated with vehicle (circles), Cell Trace Far Red (CTFR)-labeled erythrocytes (triangles) or CTFR-labeled platelets (squares). **A** After 4 h, non-internalized erythrocytes were washed away and BMDMs were stained with calcein green AM. Phagocytosis of erythrocytes (red) in BMDMs (green) was confirmed in all optical planes (xy, yz and xz) using confocal microscopy. Scale bar = 30 μm. **B**–**G** After 1, 2 and/or 4 h of incubation with vehicle, erythrocytes or platelets, BMDMs were analyzed using flow cytometry. **B** Phagocytosis was quantified by measuring CTFR positivity in BMDMs. **C** Labile iron was measured using FeRhoNox-1. **D** Lipid peroxidation was measured using Bodipy C11 581/591 and represented as the ratio of peroxidized to reduced lipids. **E** Cell death was measured using propidium iodide (PI) labeling. **F** DNA fragmentation was measured using TUNEL and expressed as fold change of mean fluorescence compared to negative control. PC = positive controls: BMDMs treated with 50 ng/mL TNFα combined with 30 µg/mL cycloheximide for 4 h. **G** BMDMs isolated from *GPX4*^*+/+*^ and *GPX4*^*Tg/+*^ mice were treated with vehicle or erythrocytes for 4 h. Cell death was measured using PI labeling. **H** BMDMs were treated with vehicle (circles), erythrocytes (triangles) or platelets (squares) for 1, 2 or 4 h. Western blot analyses of HMOX1, FTH and GPX4 were performed on cell lysates. Two-way ANOVA followed by Dunnett’s multiple comparison between vehicle and erythrocytes/platelets per timepoint (n = 4–10 independent experiments). *UT* untreated

BMDMs were treated with CTFR-labeled erythrocytes to induce erythrophagocytosis. Erythrocytes were efficiently phagocytosed by BMDMs as observed via confocal microscopy (Fig. 2A). Nearly all BMDMs contained at least one erythrocyte located in the cytosol in all optical planes (xy, yz and xz planes). Erythrophagocytosis was also confirmed and quantified via flow cytometry (Fig. 2B). A time-dependent increase in the number of BMDMs staining positive for CTFR-labeled erythrocytes was observed and reached 70% after 4 h. Similarly, BMDMs efficiently phagocytosed CTFR-labeled platelets (Fig. 2B). Erythrophagocytosis was associated with a significantly higher FeRhoNox-1 fluorescence signal as compared to vehicle-treated BMDMs at every timepoint tested, indicative of the accumulation of labile iron (Fig. 2C). This was followed by a significant increase in lipid peroxidation (Fig. 2D) and cell death (Fig. 2E) after 2 and 4 h as compared to vehicle-treated BMDMs. In contrast, platelet phagocytosis was not accompanied by an increase in labile iron, lipid peroxidation or cell death (Fig. 2C–E). The combination of increased labile iron, lipid peroxidation, and cell death are characteristic of ferroptosis, suggesting that ferroptosis is involved in erythrophagocytosis-induced cell death. No difference in TUNEL positivity was observed in BMDMs treated with erythrocytes for up to 4 h (Fig. 2F), indicating that DNA fragmentation, characteristic of apoptosis, is not involved. Surprisingly, BMDMs overexpressing GPX4 were not protected against erythrophagocytosis-induced ferroptosis (Fig. 2G). Western blot analyses showed a significant increase of HMOX1 and FTH after 4 h of erythrophagocytosis as compared to vehicle-treated BMDMs (Fig. 2H). In contrast, no changes in GPX4 expression were observed after treatment with erythrocytes, while GPX4 expression was significantly upregulated in BMDMs treated with platelets (Fig. 2H). Together, these data indicate that non-canonical rather than canonical ferroptosis is involved in erythrophagocytosis-induced cell death.

### UAMC-3203 protects BMDMs from erythrophagocytosis-induced ferroptosis and is suitable for in vivo administration via subcutaneous osmotic minipumps

**Fig. 3:**
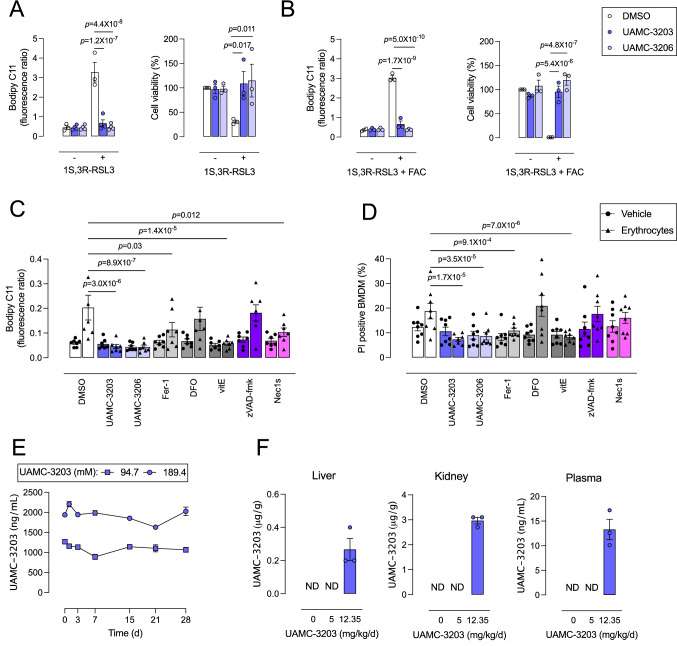
UAMC-3203 inhibits 1 S,3R-RSL3- and erythrophagocytosis-induced ferroptosis and can be administered via subcutaneous osmotic minipumps. **A**, **B** BMDMs were treated with 1 µM 1 S,3R-RSL3 alone (**A**) or combined with 100 µM ferric ammonium citrate (FAC) (**B**). After 2 h lipid peroxidation was measured with flow cytometry using Bodipy C11 581/591. After 6 h cell viability was measured using neutral red. DMSO-treated BMDMs served as 100% viability controls. Two-way ANOVA followed by Dunnett’s multiple comparison between DMSO and UAMC-3203 or UAMC-3206 (n = 3 independent experiments). **C**, **D** BMDMs were treated with vehicle (circles) or erythrocytes (triangles) combined with DMSO, 1 µM UAMC-3203, 1 µM UAMC-3206, 1 µM Ferrostatin-1 (Fer-1), 100 µM deferoxamine (DFO), 100 µM vitamin E (vitE), 20 µM zVAD-fmk or 30 µM Necrostatin-1s (Nec1s). **C** Lipid peroxidation was measured after 2 h using Bodipy C11 581/591 and represented as the ratio of peroxidized lipids to reduced lipids. **D** Cell death was measured after 4 h using propidium iodide (PI) labeling. Two-way ANOVA followed by Sidak’s multiple comparison between inhibitors and DMSO per vehicle/erythrocytes treatment (n = 8 independent experiments). **E** 94.7 mM (squares) and 189.4 mM (circles) UAMC-3203 solutions were incubated at 37 °C for 28 days. At day 0, 1, 3, 7, 15, 21 and 28, samples were taken and analyzed using LC-MS/MS (n = 3, for some points error bars are not visible). **F** Wild-type mice received 0, 5 or 12.35 mg/kg/day UAMC-3203 via subcutaneous osmotic minipumps and were sacrificed after 2 weeks (n = 3 mice per treatment group). Blood was collected and kidneys and liver were homogenized followed by UAMC-3203 measurement using LC-MS/MS. *ND* not detected

Third generation ferroptosis inhibitors UAMC-3203 and UAMC-3206 were validated in vitro in BMDMs. To induce ferroptosis, BMDMs were treated with GPX4 inhibitor 1 S,3R-RSL3 (1 µM) alone or combined with ferric ammonium citrate (FAC, 100 µM). Co-treatment with UAMC-3203 or UAMC-3206 protected BMDMs from 1 S,3R-RSL3- or 1 S,3R-RSL3/FAC-induced cell death (Fig. 3A, B). Similarly, 1 S,3R-RSL3 and 1 S,3R-RSL3/FAC induced a significant increase in lipid peroxidation, which was abrogated when BMDMs were co-treated with UAMC-3203 or UAMC-3206 (Fig. 3A, B). Thus, UAMC-3203 and UAMC-3206 inhibited 1 S,3R-RSL3- and 1 S,3R-RSL3/FAC-induced ferroptosis in BMDMs.

To investigate whether UAMC-3203 and UAMC-3206 can inhibit erythrophagocytosis-induced ferroptosis, BMDMs were treated with CTFR-labeled erythrocytes combined with cell death inhibitors. As a proof of concept, classical ferroptosis inhibitors Fer-1 (1 µM), deferoxamine (DFO, 100 µM) and vitamin E (vitE, 100 µM) as well as apoptosis inhibitor zVAD-fmk (20 µM) and necroptosis inhibitor Necrostatin-1s (Nec1s, 30 µM) were also included. None of the inhibitors significantly decreased erythrophagocytosis (data not shown). However, a significant decrease in lipid peroxidation was observed when BMDMs were co-treated with UAMC-3203, UAMC-3206, Fer-1, vitE or Nec1s as compared to DMSO (Fig. 3C). Along these lines, UAMC-3203, UACM-3206, Fer-1 and vitE significantly decreased erythrophagocytosis-induced cell death compared to DMSO (Fig. 3D). Together, these data indicate that erythrophagocytosis-induced lipid peroxidation and cell death can be inhibited by ferroptosis blockers, including the recently developed inhibitors UAMC-3203 and UAMC-3206, but not by apoptosis and necroptosis blockers.

Because UAMC-3203 and UAMC-3206 showed similar activity in vitro and UAMC-3203 has already been successfully applied in vivo in both mice and rats [10, 19, 20], only UAMC-3203 was further used for in vivo experiments. Before administering UAMC-3203 in vivo, the stability and delivery of UAMC-3203 via subcutaneous osmotic minipumps was validated. First, UAMC-3203 was dissolved in saline (0.9% NaCl) at concentrations corresponding to 28 days of low (94.70 mM solution is injected in one pump to obtain 6.18 mg/kg/day for 28 days) and high dose (189.39 mM solution is injected in one pump to obtain 12.35 mg/kg/day for 28 days) treatments in vivo, as described previously [10, 19]. To validate that both UAMC-3203 solutions are stable over a period of 28 days (corresponding to the treatment duration of one osmotic minipump), the concentration of the solutions was measured over 28 days using LC-MS/MS (Fig. 3E). The concentrations of both high and low dose solutions remained above 60% of the starting concentration and the concentration of the high dose solution even remained above 80%. Next, WT mice received 0, 5 or 12.35 mg/kg/day UAMC-3203 via subcutaneous osmotic minipumps. After 2 weeks (in the middle of the pump treatment period of 28 days), plasma, liver and kidney were collected and UAMC-3203 was measured. Interestingly, UAMC-3203 could not be detected in plasma and tissues of mice that received 5 mg/kg/day UAMC-3203 (Fig. 3F). In contrast, UAMC-3203 was detectable in plasma, kidney and liver of mice that received 12.35 mg/kg/day UAMC-3203 (Fig. 3F). In conclusion, UAMC-3203 solutions in saline are stable and administration of 12.35 mg/kg/day UAMC-3203 through subcutaneous osmotic minipumps leads to detectable UAMC-3203 levels in mice.

### FTH and HMOX1 are expressed in carotid plaques from *ApoE*^−/−^*Fbn1C1039G*^+/−^ mice

**Fig. 4:**
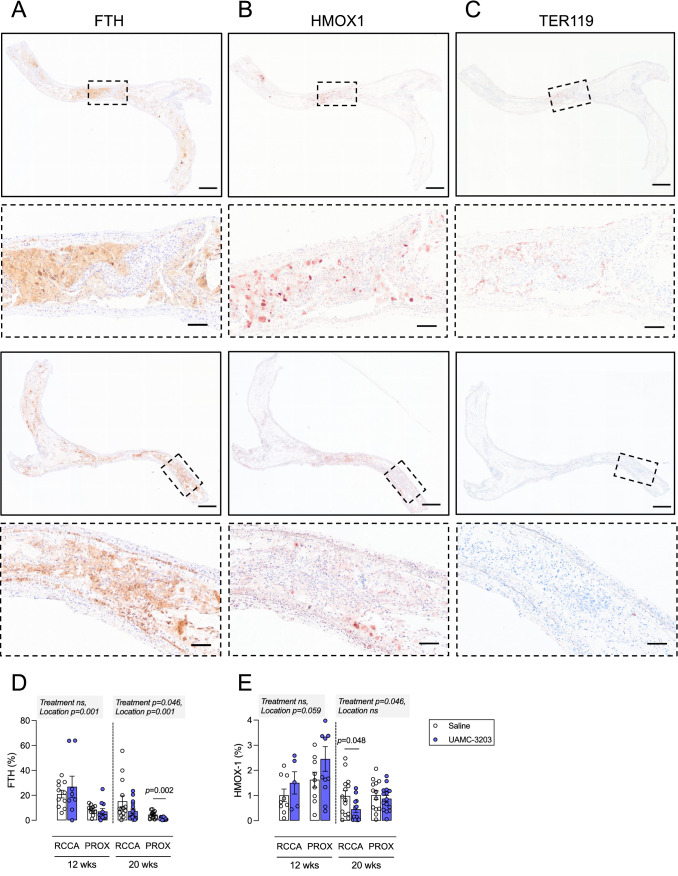
FTH, HMOX1 and TER119 are expressed in carotid plaques of *ApoE*^*−/−*^ *Fbn1*^*C1039G+/−*^ mice. **A**–**C** *ApoE*^*−/−*^ *Fbn1*^*C1039G+/−*^ mice were fed a western-type diet (WD) for 25 weeks to induce advanced plaque formation. Expression of ferritin heavy chain (FTH, A), heme-oxygenase 1 (HMOX1, B) and TER119 (C) was evaluated in serial sections of carotid lesions. Overview images, scale bar = 500 μm. Dotted frames are magnified, scale bar = 100 μm. Representative images are shown. **D**, **E** *ApoE*^*−/−*^ *Fbn1*^*C1039G+/−*^ mice were fed WD for 12 or 20 wks combined with saline (white) or UAMC-3203 (purple) treatment. Sections of the right common carotid artery (RCCA) and proximal ascending aorta (PROX) were stained with (**D**) anti-FTH and (**E**) anti-HMOX1. Independent samples *t*-test between saline and UAMC-3203 per location and time (n = 6–16 mice per group). Grey boxes: mixed-effects analysis with treatment and location as fixed effects (no significant interactions, n = 6–16 mice per group)

*ApoE* ^*−/−*^ *Fbn1*^*C1039G+/−*^ mice develop advanced atherosclerotic plaques with intraplaque angiogenesis, hemorrhages and possibly erythrophagocytosis [14, 15]. Because erythrophagocytosis increases expression levels of HMOX1 and FTH in BMDMs in vitro, immunohistochemical analysis of HMOX1 and FTH was performed on plaques from *ApoE*^*−/−*^ *Fbn1*^*C1039G+/−*^ mice. Because the latter mouse model develops advanced plaques with IP angiogenesis and plaque rupture in the carotid artery [15], we focused our analyses on carotid lesions. To enable evaluation of IP angiogenesis, longitudinal sections were used. FTH positivity was abundantly present across the lesion (Fig. 4A), especially in areas positive for HMOX1 (Fig. 4B). In the same areas, TER119 positivity was also observed, indicating that IP erythrocytes are present (Fig. 4C). Importantly, carotid lesions with confirmed IP angiogenesis always revealed IP hemorrhages, supporting previous data that IP neovessels are fragile and leaky [15]. Since IP hemorrhages, unlike IP angiogenesis, are relatively easy to detect via histology, 20% (8 of 40) carotid lesions showed IP hemorrhages without confirmed IP angiogenesis. If IP hemorrhages were not detectable in carotid lesions, IP angiogenesis was not visible either.

**Fig. 5:**
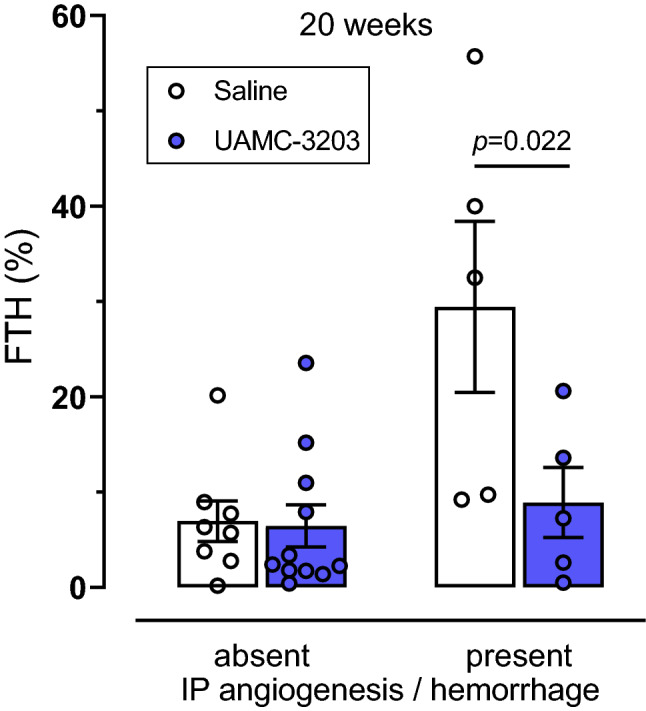
Overexpression of ferritin heavy chain (FTH) in carotid plaques from *ApoE*^*−/−*^ *Fbn1*^*C1039G+/−*^ mice after prolonged western-type diet (WD) can be prevented with ferroptosis inhibitor UAMC-3203. *ApoE*^*−/−*^ *Fbn1*^*C1039G+/−*^ mice were fed WD for 20 weeks combined with saline (white) or UAMC-3203 (purple) treatment. Sections of the right common carotid artery (RCCA) with or without confirmed IP angiogenesis/hemorrhage were stained with anti-FTH and percentage immunoreactivity was quantified. Two way ANOVA (n = 5–11 mice per group)

To evaluate the effect of ferroptosis inhibition on atherogenesis, *ApoE*^*−/−*^ *Fbn1*^*C1039G+/−*^ mice were fed a WD combined with UAMC-3203 administered via subcutaneous osmotic minipumps. To distinguish effects associated with IP angiogenesis and hemorrhages from other mechanisms, *ApoE*^*−/−*^ *Fbn1*^*C1039G+/−*^ mice were randomly divided over two treatment regimens, a shorter protocol covering 12 weeks of WD (IP angiogenesis is not occurring yet, or recently initiated) and a longer protocol up to 20 weeks of WD (IP angiogenesis frequently occurring in carotid plaques from *ApoE*^*−/−*^ *Fbn1*^*C1039G+/−*^ mice). In both treatment regimens, mice received UAMC-3203 (12.35 mg/kg/d) during the last 8 weeks of the protocol (starting at week 4 or 12) via two sequential osmotic minipumps, each covering a period of 4 weeks. Similar to what was observed after erythrophagocytosis in vitro and in erythrocyte-rich murine lesions, FTH and HMOX1 were expressed after 12 and 20 weeks WD in carotid plaques from *ApoE*^*−/−*^ *Fbn1*^*C1039G+/−*^ mice (Fig. 4D, E). Both FTH and HMOX1 were also expressed in plaques in the proximal ascending aorta. Interestingly, UAMC-3203 treatment significantly decreased expression of FTH and HMOX1 expression after 20 weeks WD in carotid and proximal aorta plaques. These effects were not observed after 12 weeks WD (Fig. 4D, E). If we examine carotid plaques after 20 weeks WD with and without IP hemorrhages separately, FTH expression significantly increased in plaques with confirmed IP hemorrhages and this effect was blocked by UAMC-3203 (Fig. 5) HMOX1 expression also increased in plaques with confirmed IP hemorrhages (0.8 ± 0.3% in plaques without IP hemorrhages; 5.3 ± 4.3% in plaques with confirmed IP hemorrhages), but the overexpression was not inhibited by UAMC-3203 (data not shown).

### UAMC-3203 limits atherogenesis in the carotid artery from *ApoE*^−/−^*Fbn1*^C1039G+/−^ mice after 20 weeks WD

**Fig. 6:**
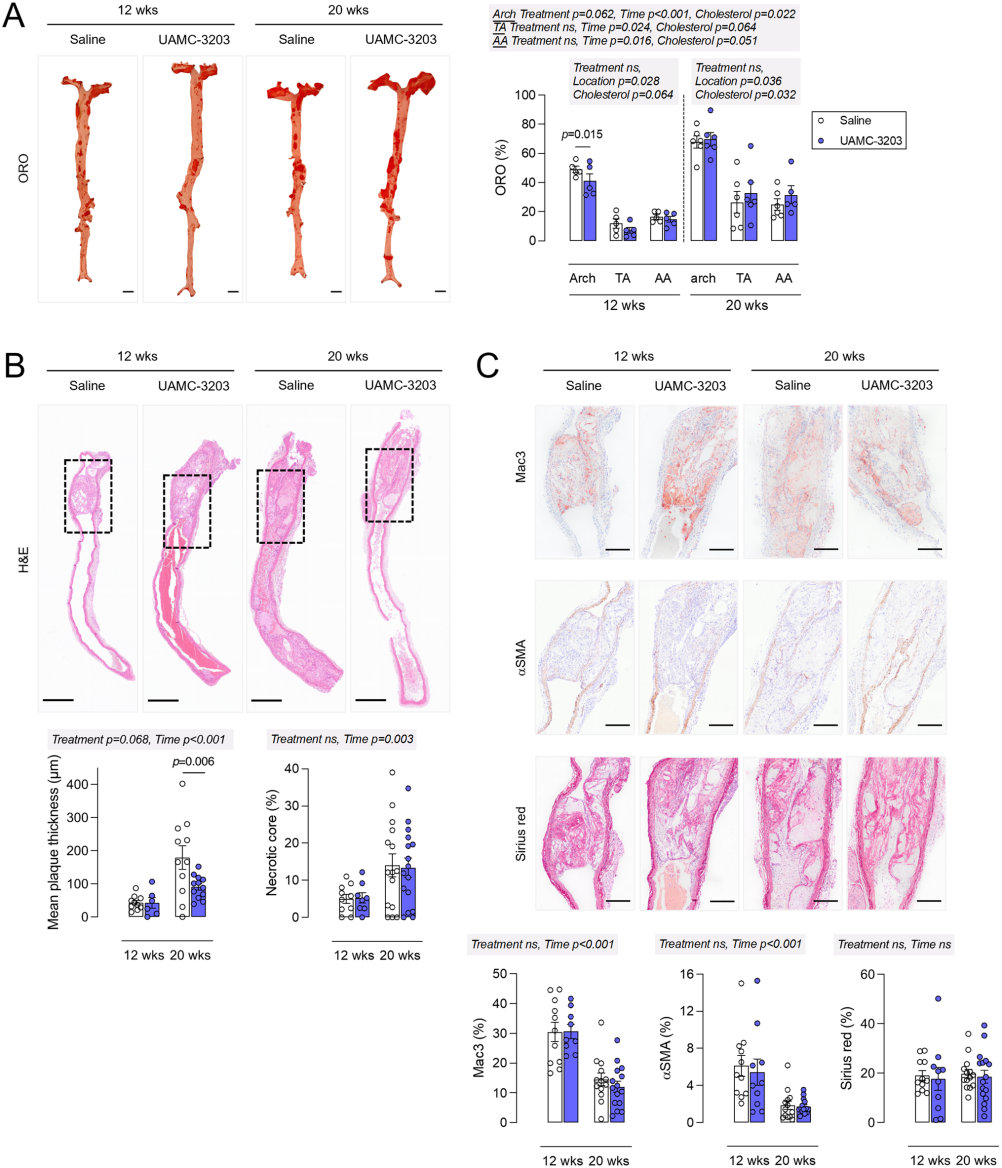
Plaque analysis of *ApoE*^*−/−*^ *Fbn1*^*C1039G+/−*^ mice fed a western-type diet and treated with UAMC-3203 or saline. *ApoE*^*−/−*^ *Fbn1*^*C1039G+/−*^ mice were fed a WD for 12 or 20 wks combined with saline (white) or UAMC-3203 (purple) treatment. **A** The aortic arch, thoracic (TA) and abdominal (AA) aorta were stained *en face* with Oil Red O to determine lipid burden. Top grey box: two-way ANOVA with simple main effects analysis of treatment and time for arch, TA or AA separately (plasma cholesterol was included as covariate, no significant interactions, n = 5–6 mice per group). Two bottom grey boxes: two-way ANOVA with repeated measures: location as within-subjects variable and treatment as between-subjects variable, single main effects analysis for 12 or 20 wks WD separately (plasma cholesterol was included as covariate, no significant interactions, n = 4–6 mice per group). Scale bar = 5 mm. **B** Sections of the right common carotid artery (RCCA) were stained with hematoxylin/eosin to quantify the mean plaque thickness over the whole RCCA and necrotic cores. Data of all mice are shown (i.e., no classification between plaques with or without IP angiogenesis or hemorrhage). Two-way ANOVA with simple main effects analysis (no interactions, plasma cholesterol was included as covariate but was never significant, n = 5–16 mice per group). Scale bar = 500 μm. **C** Sections of the RCCA were stained with anti-Mac3 to determine macrophage content, anti-α-smooth muscle actin (α-SMA) to determine vascular smooth muscle cell content and Sirius Red to determine collagen content. Dotted frames in images of (**B**) correspond to same regions in magnified images of (**C**). Scale bar = 200 μm. Two-way ANOVA with simple main effects analysis (plasma cholesterol was always included as covariate but was never significant, no significant interactions, n = 6–16 mice per group). Representative images are shown

**Fig. 7:**
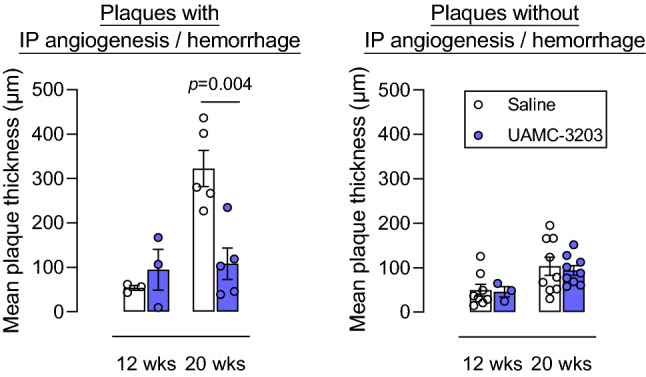
Enhanced plaque formation that only occurs in *ApoE*^*−/−*^ *Fbn1*^*C1039G+/−*^ mice after IP angiogenesis and/or hemorrhages and prolonged (20 weeks) western-type diet can be prevented with ferroptosis inhibitor UAMC-3203. *ApoE*^*−/−*^ *Fbn1*^*C1039G+/−*^ mice were fed a WD for 12 or 20 wks combined with saline (white) or UAMC-3203 (purple) treatment. Sections of the right common carotid artery (RCCA) from mice were stained with hematoxylin/eosin to quantify the mean plaque thickness over the whole RCCA. Data of mice with (left hand panel) or without (right hand panel) IP angiogenesis or hemorrhages in the RCCA are shown. Independent samples *t*-test between saline and UAMC-3203 (n = 3–9 mice per group)

UAMC-3203 treatment did not alter survival rates compared to saline treatment (data not shown). No differences in body weight were present between the treatment groups (25.0 ± 0.7 vs. 24.2 ± 0.7 g after 12 weeks WD and 25.7 ± 0.6 vs. 25.5 ± 0.5 g after 20 weeks WD, *p* > 0.05). A minimal decrease in plasma cholesterol levels was observed in UAMC-3203-treated mice after both 12 weeks WD (488 ± 18 vs.433 ± 20 mg/dl, *p* = 0.025) and 20 weeks WD (489 ± 17 vs. 435 ± 17 mg/dl, *p* = 0.038). The aortic arch, thoracic (TA) and abdominal (AA) aorta were dissected and stained *en face* with Oil Red O (ORO) to determine lipid burden (Fig. 6A). Over the entire aorta lipid burden was not significantly changed in UAMC-3203-treated mice as compared to saline-treated controls after 12 and 20 weeks WD (two-way ANOVA with simple main effects analysis, treatment effect *p* = 0.329, time effect *p* = 0.002, interaction *p* = 0.086, cholesterol effect *p* = 0.024). There was no overall treatment effect on lipid burden in the aortic arch. However, UAMC-3203 significantly decreased ORO positivity in the aortic arch after 12 weeks WD but not after 20 weeks (two-way ANOVA with simple main effects analysis, 12 weeks WD *p* = 0.015, 20 weeks WD *p* = 0.972, but interaction was borderline not-significant *p* = 0.053). In TA and AA, no significant changes of lipid burden were observed between treatment groups. Similarly, when aorta location was considered as a repeated measure, no significant effects of UAMC-3203 treatment were observed in the arch, TA and AA after 12 or 20 weeks WD. In conclusion, although a small decrease in lipid burden was observed in the aortic arch from UAMC-3203-treated mice as compared to saline-treated controls after 12 weeks WD, in general UAMC-3203 treatment did not significantly alter atherogenesis in the thoracic, abdominal and entire aorta from *ApoE*^*−/−*^ *Fbn1*^*C1039G+/−*^ mice after 12 and 20 weeks WD. Likewise, plaque area was not changed in the proximal ascending aorta from UAMC-3203-treated *ApoE*^*−/−*^ *Fbn1*^*C1039G+/−*^ mice as compared to saline-treated controls (data not shown). In contrast, the mean plaque thickness in the carotid artery was significantly decreased after 20 weeks WD in UAMC-3203-treated mice as compared to controls (Fig. 6B, two-way ANOVA with simple main effects analysis, 12 weeks WD *p* = 0.958, 20 weeks WD *p* = 0.006; Independent samples *t*-test, 20 weeks WD *p* = 0.035). When only carotid plaques with confirmed IP angiogenesis and/or hemorrhage (identified with anti-TER119 immunostaining, respectively) were included, the difference in mean plaque thickness in saline-treated versus UAMC-3203-treated mice after 20 weeks WD was even more clear (Fig. 7). Importantly, this effect was not yet present after 12 weeks WD and in plaques without IP hemorrhage (Fig. 7). Obviously, necrotic cores were significantly larger after 20 weeks WD compared to 12 weeks WD. However, UAMC-3203 treatment had no effect on the relative necrotic core areas as compared to controls (Fig. 6B). In addition, no differences in macrophage, vascular smooth muscle cell and collagen content were observed in UAMC-3203-treated mice as compared to saline-treated controls after both 12 and 20 weeks WD (Fig. 6C). Also further analysis of the composition of plaques with or without intraplaque angiogenesis/hemorrhage and treated with saline or UAMC-3203 did not reveal statistically significant differences, neither after 12 weeks WD nor after 20 weeks WD (Table 1). In conclusion, while plaque formation in the thoracic and abdominal aorta was not affected, inhibition of ferroptosis with UAMC-3203 decreased plaque thickness in the carotid artery from *ApoE*^*−/−*^ *Fbn1*^*C1039G+/−*^ mice after 20 weeks WD, but not in earlier stages of plaque progression after 12 weeks WD and in plaques without IP hemorrhage.

**Table 1:** Composition of atherosclerotic plaques with or without intraplaque angiogenesis/hemorrhage in *ApoE*^*-/-*^ *Fbn1*^*C1039G+/-*^ mice treated with saline or UAMC-3203 in combination with WD for 12 or 20 weeks

		12 wks WD		20 wks WD	
	IP angiogenesis / hemorrhage	Saline	UAMC- 3203	Saline	UAMC- 3203
Necrotic core (%)	+	3.5 ± 1.3	5.6 ± 3.6	14.7 ± 3.8	10.3 ± 4.6
	−	5.4 ± 1.5	5.9 ± 1.9	14.3 ± 5.5	15.1 ± 3.5
Mac3 (%)^a^	+	32.9 ± 8.4	28.6 ± 3.8	16.9 ± 4.3	10.5 ± 4.6
	−	29.5 ± 3.6	30.0 ± 5.8	13.0 ± 3.2	13.8 ± 1.5
αSMA (%)^b^	+	5.0 ± 1.8	7.5 ± 4.1	2.0 ± 1.1	2.3 ± 0.4
	−	6.5 ± 1.4	6.4 ± 2.5	1.6 ± 0.4	1.5 ± 0.2
Sirius red (%)^c^	+	18.7 ± 5.3	22.1 ± 14.1	16.7 ± 2.4	12.5 ± 4.0
	−	19.2 ± 2.1	21.3 ± 2.9	20.9 ± 2.7	19.5 ± 2.8

Data from the right common carotid artery, mean ± SEM

Data are not statistically different between saline and UAMC-3203 (Independent samples *t*-test, n = 3–10 mice per group)

^a^Marker of macrophage content

^b^Marker of vascular smooth muscle cell content

^c^Marker of collagen content

## Discussion

A direct association between IP angiogenesis/hemorrhage, plaque progression and necrotic core volume was previously observed in human carotid lesions using high-resolution magnetic resonance imaging [21]. Moreover, IP hemorrhage in human carotid plaques is associated with an increased stroke risk [22, 23], and has been proposed as an imaging marker to predict future thrombosis events (in the same or other vascular sites) [24]. Thus, IP angiogenesis and hemorrhage clearly contribute to human plaque progression and instability [1, 2], even though the exact mechanisms are not clear-cut. IP hemorrhage promotes infiltration of erythrocytes and platelets in the plaque region. Once inside the plaque, erythrocytes undergo oxidative damage, which results in the formation of lipid-protein conjugates closely related to some of the conjugates found in oxidized LDL [25]. In this way, oxidized erythrocytes become ligands for the macrophage scavenger receptors and are rapidly cleared by phagocytosis. Clearance of platelets by macrophages is mediated through different types of receptors including class A scavenger receptors, the phosphatidylserine receptor and CD36 [26]. After phagocytosis of erythrocytes or platelets, macrophages become activated as shown by elevated activity of inducible NO synthase (iNOS), and produce autofluorescent pigment with the characteristics of ceroid, an insoluble mixture of oxidized lipids and proteins that can be found frequently in foam cells around neovessels in human plaques [3, 4]. Erythrophagocytosis is also associated with impaired uptake of apoptotic cells (efferocytosis) [27], which may promote necrotic core formation. Of note, IP hemorrhage and erythrophagocytosis is characterized by the presence of erythrocyte-derived heme iron in the vicinity of necrotic cores [1–4, 28]. Therefore, we hypothesized that erythrophagocytosis induces ferroptosis, which may further contribute to growth of the necrotic core and hence, plaque progression and destabilization. In the present study, we mimicked these processes in vitro by treating bone marrow-derived macrophages (BMDMs) with erythrocytes or platelets. First, we confirmed that BMDMs readily phagocytose platelets and erythrocytes in our in vitro set-up. The fast in vitro clearance of erythrocytes by macrophages was intriguing as untreated erythrocytes usually do not allow efficient binding and phagocytosis by macrophages. However, we used aged erythrocytes from recently expired human erythrocyte concentrates that may be cleared quite rapidly by macrophages based on changes in membrane carbohydrates, exposure of phosphatidylserine and oxidative damage [29]. Erythrophagocytosis resulted in significant cell death while platelet phagocytosis did not. Both platelet and erythrophagocytosis contribute to lipid-enrichment and foam cell formation [3, 4]. However, platelets do not contain high levels of iron suggesting that iron, rather than lipids, is crucial in erythrophagocytosis-induced cell death. Indeed, erythrophagocytosis induced an increase of the labile iron pool followed by an increase of lipid peroxidation, which are hallmark features of ferroptosis. Co-treatment of BMDMs with ferroptosis inhibitors UAMC-3203, UAMC-3206, Fer-1 or vitamin E significantly reduced erythrophagocytosis-induced lipid peroxidation and cell death, while apoptosis and necroptosis inhibitors did not, which further confirms that erythrophagocytosis induces ferroptosis. These observations are in line with previous experiments showing that enhanced erythrophagocytosis, induced by treatment of murine and human macrophages with IgG-coated erythrocytes, resulted in increased lipid peroxidation and cell death, which was abrogated by Fer-1 [30]. Likewise, erythrophagocytosis of mutant (*JAK2*^*V617F*^ mutation, which enhances erythrophagocytosis) or normal erythrocytes also resulted in ferroptosis, which was abrogated by the ferroptosis inhibitor liproxstatin-1 [31].

Western blot analyses showed a significant upregulation of HMOX1 and FTH expression in BMDMs after erythrophagocytosis. Along the same lines, previous experiments demonstrated that erythrophagocytosis of normal, mutant (*JAK2*^*V617F*^) or UV-oxidized erythrocytes results in increased FTH levels [11, 31]. Likewise, increased HMOX1 expression was described in plaque macrophages surrounding hemorrhages [4], in splenic red pulp macrophages after erythrophagocytosis [30], and during ferroptosis in high fat and high glucose-treated endothelial cells [32], and in erastin-treated vascular smooth muscle cells [33]. Together, these findings indicate that following erythrophagocytosis, heme (from engulfed erythrocytes) is catabolized by HMOX1 to biliverdin, carbon monoxide and ferrous iron, which is oxidized and stored by FTH. We observed a strong increase in HMOX1 expression (approximately 10-fold) after erythrophagocytosis, suggesting excessive activation of HMOX1 and production of huge amounts of ferrous iron, possibly beyond the buffering capacities of FTH, which is sufficient to induce non-canonical ferroptosis [7]. Furthermore, in the present study, no change in expression of GPX4 was observed after erythrophagocytosis and GPX4 overexpression in BMDMs was not protective against cell death, which indicates that ferroptosis is not initiated through a canonical (GPX4-mediated) pathway. Interestingly, a significant increase of GPX4 expression was detected in BMDMs treated with platelets in the absence of cell death, lipid peroxidation or increased labile iron. Both platelets and erythrocytes express GPX4 and hence, their phagocytosis may contribute to increased GPX4 expression levels in BMDM lysates. Platelet phagocytosis was > 95% efficient, meaning that almost all BMDMs had engulfed platelets (in contrast to only 70% erythrophagocytosis), which may provide an additional explanation for the significant increase of GPX4 expression in BMDM lysates after platelet phagocytosis.

UAMC-3203 and UAMC-3206 are novel, third generation Fer-1 derivates with improved pharmacokinetic properties and increased solubility and potency [10]. Both compounds differ only in their solubility-increasing moiety, with UAMC-3203 containing a piperazine and UAMC-3206 an amine. They show similar potency and solubility, but UAMC-3203 was reported to show higher microsomal stability in humans and rats and was therefore preferred over UAMC-3206 for in vivo experiments [10, 19]. Moreover, an optimized and validated method for UAMC-3203 (but not UAMC-3206) detection was available and the in vivo dosing, stability and toxicity of UAMC-3203 in mice was previously described [10, 19, 20]. To evaluate the in vivo delivery of UAMC-3203 via subcutaneous osmotic minipumps, we administered 0, 5 or 12.35 mg/kg/day UAMC-3203 through osmotic minipumps in WT mice. A dose of 5 mg/kg/day UAMC-3203 did not result in detectable UAMC-3203 levels in plasma, kidney or liver after 2 weeks, which contrasts with what was reported after intravenous administration of similar dosages in rats [10, 19]. This discrepancy can be explained by lower microsomal stability (t_1/2_ = 3.46 ± 1.37 h vs. 16.48 ± 4.66 h) and higher clearance (Cl_int_ = 18.0 ± 7.1 vs. 3.4 ± 1.0 mL/min/kg) of UAMC-3203 in mice as compared to rats [10]. However, administration of 12.35 mg/kg/day UAMC-3203 resulted in detectable UAMC-3203 levels in plasma, kidney and liver, and was therefore used for further in vivo experiments.

To evaluate erythrophagocytosis and ferroptosis in atherosclerosis, the *ApoE*^*−/−*^ *Fbn1*^*C1039G+/−*^ mouse model was applied. In contrast to classical *ApoE*^*−/−*^ and *LDLr*^*−/−*^ mice, *ApoE*^*−/−*^ *Fbn1*^*C1039G+/−*^ mice develop advanced atherosclerotic plaques with IP angiogenesis and hemorrhage in the carotid artery [14, 15], which is obviously required to tackle erythrophagocytosis-induced effects in atherosclerosis in vivo. First, we confirmed that erythrocytes are present in carotid lesions of *ApoE*^*−/−*^ *Fbn1*^*C1039G+/−*^ mice. Similar to what was observed in vitro after erythrophagocytosis, FTH and HMOX1 were also expressed in carotid plaques of *ApoE*^*−/−*^ *Fbn1*^*C1039G+/−*^ mice, which is in line with previous reports demonstrating that HMOX1 and ferritin accumulate in advanced human plaques [4, 11, 33]. Moreover, in the present study, HMOX1 and ferritin were present in erythrocyte-rich regions of carotid plaques, which provides a link between IP hemorrhage and ferroptosis. Next, we evaluated the effect of ferroptosis inhibition with UAMC-3203 on atherosclerosis in *ApoE*^*−/−*^ *Fbn1*^*C1039G+/−*^ mice after 12 weeks of WD, when IP angiogenesis is not yet occurring (or at least not for a long time), and after 20 weeks of WD, when plaques show established IP angiogenesis and hemorrhage [14, 15, 34]. With this study design, we aimed to distinguish effects of ferroptosis inhibition associated with IP hemorrhage and erythrocytes from other mechanisms. Indeed, beneficial effects of ferroptosis inhibition on atherosclerosis may not be linked to IP hemorrhage alone. For example, two studies reported reduced plaque burden in the aorta after ferroptosis inhibition with Fer-1 in high fat (and high glucose)-fed *ApoE*^*−/−*^ mice (a model that does not frequently develop IP angiogenesis [15]), which was linked to attenuated endothelial dysfunction [32, 35].

Although a small decrease in plasma cholesterol was observed between saline- and UAMC-3203-treated groups, mean plasma cholesterol levels always remained above 400 mg/dL in UAMC-3203-treated mice. Nevertheless, plasma cholesterol concentrations were included as covariate in ANOVA analyses, showing that the modest decrease in plasma cholesterol did not affect plaque parameters in the proximal aorta and carotid artery. Analysis of the lipid burden in the thoracic and abdominal aorta of saline- and UAMC-3203-treated *ApoE*^*−/−*^ *Fbn1*^*C1039G+/−*^ mice revealed no differences between treatment groups after 12 and 20 weeks WD. Similarly, no differences in plaque area were observed in the proximal ascending aorta. However, we observed a significant decrease in mean plaque thickness in the carotid artery from UAMC-3203-treated mice as compared to saline after 20 weeks WD, but not after 12 weeks WD. These results suggests that inhibition of ferroptosis mainly affects plaques with a more advanced phenotype and established IP angiogenesis and hemorrhage, which may not be the case in carotid plaques at early stages (i.e. 12 weeks WD) and in aortic plaques [15]. Further evidence for this assumption comes from mice with confirmed IP angiogenesis or hemorrhage after 20 weeks WD (based on immunohistochemical analysis of Ter119) demonstrating a more pronounced decrease in plaque thickness after UAMC-3203 treatment as compared to plaques from control mice. Reciprocally, when only considering plaques without visually confirmed IP angiogenesis or hemorrhage, no significant difference in carotid plaque thickness was observed. Interestingly, thickness of carotid plaques from saline-treated controls without IP hemorrhage were approximately 3-fold smaller than from saline-treated controls with IP hemorrhage, which supports the statement that IP hemorrhage contributes to plaque progression [1, 2]. UAMC-3203 treatment significantly decreased FTH and HMOX1 expression in plaques of the carotid artery and proximal aorta from *ApoE*^*−/−*^ *Fbn1*^*C1039G+/−*^ mice after 20 weeks WD, but not after 12 weeks. HMOX-1 is a stress-responsive enzyme that can be activated by oxidative stress through the antioxidant responsive element (ARE). UAMC-3203, on the other hand, functions as a radical trapping agent in lipid bilayers [9, 10], thus UAMC-3203 takes away a stress factor which may explain the observed decrease in HMOX-1 expression in plaques from UAMC-3203-treated mice. Consequently, it is plausible that less hemoglobin is degraded to ferrous iron, which in turn may explain the observed decrease in FTH expression. Indeed, it was previously reported that decreased HMOX1 expression (induced by treatment with HMOX1-specific siRNA) decreases iron content and lipid peroxidation, and thus ferroptosis, in aortic endothelial cells from diabetic *ApoE*^*−/−*^ mice [32]. Together, these observations suggest that UAMC-3203 treatment limited ferroptosis in plaques with established IP angiogenesis and hemorrhage.

Whether UAMC-3203 only affects plaque macrophages or also erythrocytes that infiltrated the plaque via IP angiogenesis/hemorrhage is currently unclear. Because erythrocytes rapidly undergo lipid peroxidation followed by phagocytosis via macrophages, it is plausible to assume that UAMC-3203 inhibits both oxidation of erythrocytes, an important prerequisite to be recognized by macrophages, and induction of macrophage ferroptosis after erythrophagocytosis. Our in vitro data did not reveal differences in erythrophagocytosis after treatment with UAMC-3203, indicating that phagocytosis as such is not affected by UAMC-3203. Remarkably, UAMC-3203 treatment had no effect on the size of necrotic cores as compared to controls, which might indicate that ferroptosis contributes only to a very limited extent to necrotic core formation in atherosclerosis. Recent studies have shown that several other forms of regulated necrosis such as pyroptosis, necroptosis and apoptosis-mediated secondary necrosis play a prominent role in the development of a necrotic core in advanced plaques [36]. Moreover, it should be noted that necrosis inhibitors have no impact on a necrotic core that has already formed.

## Conclusions

Erythrophagocytosis induced non-canonical ferroptosis in vitro which was characterized by increased HMOX1 and FTH expression. In line with this finding, HMOX1 and FTH expression were observed in erythrocyte-rich plaque regions from *ApoE*^*−/−*^ *Fbn1*^*C1039G+/−*^ mice, suggesting involvement of erythrophagocytosis-induced non-canonical ferroptosis in atherosclerosis. UAMC-3203 treatment decreased intraplaque FTH and HMOX1 levels and limited plaque progression in the carotid artery from *ApoE*^*−/−*^ *Fbn1*^*C1039G+/−*^ mice after 20 weeks WD, especially in plaques with confirmed intraplaque hemorrhage. In contrast, changes in plaque progression were not observed in earlier stages of plaque development (after 12 weeks WD) or in plaques without established hemorrhage. These results show that erythrophagocytosis-induced ferroptosis is involved in advanced stages of plaque progression and can be specifically targeted with UAMC-3203. Altogether, our study provides insight into the mechanisms contributing to plaque destabilization induced by IP angiogenesis and hemorrhage and opens doors to novel plaque stabilizing therapeutic strategies.

## Data Availability

The authors declare that all supporting data are available within the article.
